# Asymmetric expression of homoeologous genes contributes to dietary adaption of an allodiploid hybrid fish derived from *Megalobrama amblycephala* (♀) × *Culter alburnus* (♂)

**DOI:** 10.1186/s12864-021-07639-6

**Published:** 2021-05-19

**Authors:** Wuhui Li, Shi Wang, Jie Hu, Chenchen Tang, Chang Wu, Junmei Liu, Li Ren, Chengfei Sun, Junjian Dong, Shaojun Liu, Xing Ye

**Affiliations:** 1 State Key Laboratory of Developmental Biology of Freshwater Fish, Hunan Normal University, Changsha, 410081 Hunan China; 2 Key Laboratory of Tropical and Subtropical Fishery Resource Application and Cultivation of Ministry of Agriculture and Rural Affairs, Pearl River Fisheries Research Institute, Chinese Academy of Fishery Sciences, Guangzhou, 510380 Guangdong China

**Keywords:** Allodiploid hybrid, Homoeologous genes, Subgenome expression dominance, Dietary adaption

## Abstract

**Background:**

Hybridization, which can quickly merge two or more divergent genomes and form new allopolyploids, is an important technique in fish genetic breeding. However, the merged subgenomes must adjust and coexist with one another in a single nucleus, which may cause subgenome interaction and dominance at the gene expression level and has been observed in some allopolyploid plants. In our previous studies, newly formed allodiploid hybrid fish derived from herbivorous *Megalobrama amblycephala* (♀) × carnivorous *Culter alburnus* (♂) had herbivorous characteristic. It is thus interesting to further characterize whether the subgenome interaction and dominance derive dietary adaptation of this hybrid fish.

**Results:**

Differential expression, homoeolog expression silencing and bias were investigated in the hybrid fish after 70 days of adaptation to carnivorous and herbivorous diets. A total of 2.65 × 10^8^ clean reads (74.06 Gb) from the liver and intestinal transcriptomes were mapped to the two parent genomes based on specific SNPs. A total of 2538 and 4385 differentially expressed homoeologous genes (DEHs) were identified in the liver and intestinal tissues between the two groups of fish, respectively, and these DEHs were highly enriched in fat digestion and carbon metabolism, amino acid metabolism and steroid biosynthesis. Furthermore, subgenome dominance were observed in tissues, with paternal subgenome was more dominant than maternal subgenome. Moreover, subgenome expression dominance controlled functional pathways in metabolism, disease, cellular processes, environment and genetic information processing during the two dietary adaptation processes. In addition, few but sturdy villi in the intestine, significant fat accumulation and a higher concentration of malondialdehyde in the liver were observed in fish fed carnivorous diet compared with fish fed herbivorous diet.

**Conclusions:**

Our results indicated that diet drives phenotypic and genetic variation, and the asymmetric expression of homoeologous genes (including differential expression, expression silencing and bias) may play key roles in dietary adaptation of hybrid fish. Subgenome expression dominance may contribute to uncovering the mechanistic basis of heterosis and also provide perspectives for fish genetic breeding and application.

**Supplementary Information:**

The online version contains supplementary material available at 10.1186/s12864-021-07639-6.

## Background

Hybridization can quickly merge two or more divergent genomes to form new allopolyploids and is a driving force of genomic evolution and speciation [1]. The merged subgenomes must adjust and coexist with one another in a single nucleus, frequently causing genetic incompatibilities that induce a series of rapid genetic and epigenetic modifications [2]. These modifications include sequence elimination, activation of genes and retroelements, homoeologous interactions and exchanges, which may directly affect the establishment of a nascent polyploid and its evolutionary success as a new species [3–5]. An observation that may be easily linked to the success of newly formed polyploids is the variations in the expression of homeologous genes, including differential expression and subgenome dominance (one of the parental subgenomes having retained more genes and being more highly expressed, including expression silencing and bias), which have been observed in some allopolyploid plants and a few animals [2–8]. Subgenome dominance contributing to partitioning of certain phenotypic traits has been reported in cotton (*Gossypium*) [9], wheat (*Triticum aestivum*) [10, 11], maize (*Zea mays ssp. mays L*) [12] and blueberry (*Vaccinium corymbosum*) [13].

In fish, as in other vertebrates, genetic variability and phenotypic plasticity could promote within-species or within-population diversity of individual foraging strategies, while dietary shift (adaption) is likely to enhance the overall phenotypic variance and additive genetic evolution in fish populations [14–17]. Genetic variability, including genome evolution (genetic variation, gene duplication, gene family expansion and contraction, allopolyploidy, etc.) and gene regulation (epigenetic), contributing to feeding behavior and dietary adaptation, have been documented in humans [18], dogs [19], giant panda (*Ailuropoda melanoleuca*) [20], bats [21] and fishes [7, 22, 23]. Recently, growing evidences has shown that feeding also drives phenotype and genetic evolution, which has been demonstrated in perch (*Perca fluviatilis* L.) [15], stickleback (Teleostei: Stichaeidae) [24], and baleen whales (*Llanocetus denticrenatus*) [25]. However, the genotype × diet interactions contributing to foraging strategies in fish are poorly studied, especially those newly formed hybrid fish species.

Hybridization is an important technique and is widely used in fish genetic breeding. In our previous study, we obtained a hybrid lineage fish derived from herbivorous blunt snout bream (*Megalobrama amblycephala*, BSB, ♀) × carnivorous topmouth culter (*Culter alburnus*, TC, ♂) [26]. The hybrid fish are bisexually fertile and contain one set of chromosomes from each parent, providing an idea module to investigate interesting biological processes. A comparative analysis of the gut micorbiota communities and gastrointestinal tract between the hybrid and parents suggested that hybrid fish are biased toward herbivores [27]. A recent study further observed asymmetric allelic expression patterns (including additive and dominant effects and cis and trans regulations) in the hybrid lineages based on the whole-genome sequences of BSB and TC [28]. These results provide new insights into alternative strategies for counteracting deleterious effects of subgenomes and improving the adaptability of novel hybrids. It would thus interesting to further characterize whether the subgenome interaction derives dietary adaptation of the hybrid fish. In this study, we comparatively analyzed the intestinal and liver transcriptomes of hybrid fish after 70 days adaptation to herbivorous and carnivorous diets. We focus on the differentially expressed homoeologous genes, as well as the subgenome interaction (homoeolog expression silencing and bias), between the two groups of fish. This study may provide basic data for investigation of the signal transduction pathways that mediate food digestion, metabolism and adaptation and also may provide perspectives for fish breeding and application.

## Results

### Histological study of the intestine and liver

**Fig. 1:**
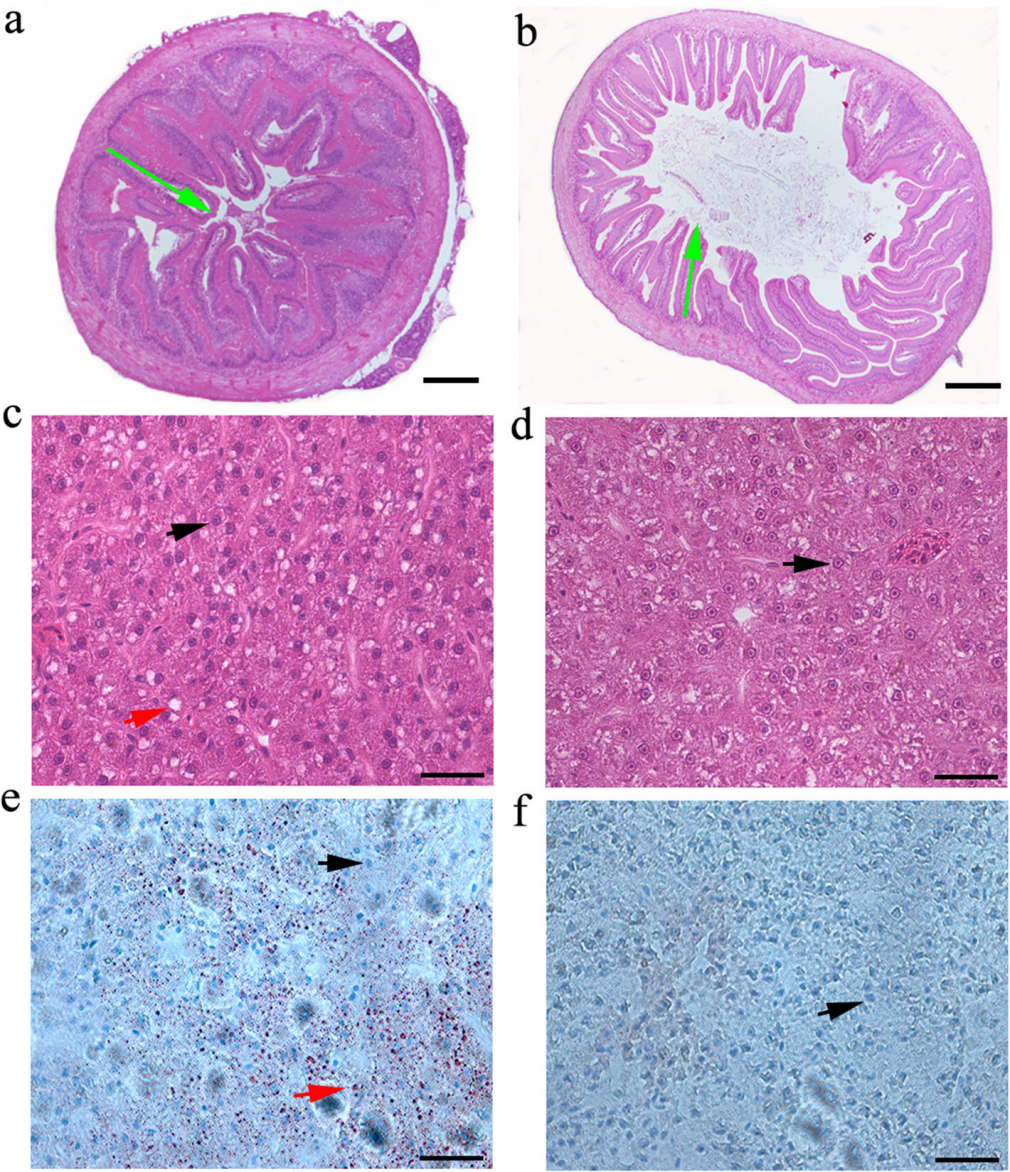
Representative photomicrographs of the intestinal and liver tissue in hybrid fish fed carnivorous and herbivorous diets. Intestinal tissue from fish fed carnivorous diet **a** herbivorous diet **b**. Liver tissue from fish fed carnivorous diet herbivorous diet, stained with H&E **c** and **d** and Oil red O **e** and **f**. The green arrow shows the intestinal villi, the black arrow shows the hepatocytes nucleus, and the red arrow shows the fat cells and lipid droplets

Histological analysis showed a significant difference in the morphology of intestinal and liver tissues in the two dietary groups of hybrid fish. The intestinal villi in fish fed carnivorous diet were sturdy but few in number than those in fish fed herbivorous diet (fine and abundant villi) (Fig. 1a, b). Normal hepatocytes with regular, round nuclei were observed in the livers of both two groups of fish. However, a small amount of hypertrophic hepatocytes lacked nuclei in fish fed herbivorous diet, while a large number of lipid droplets in the liver of the fish fed carnivorous diet were clearly observed (Fig. 1c, d). In addition, significant fat accumulation in the liver tissues of fish fed carnivorous diet was confirmed by Oil red O staining method (Fig. 1e, f).

### Differentially expressed homoeologous genes associated with dietary adaptation

A total of 2.65 × 10^8^ clean reads (74.06 Gb) were obtained from the intestinal and liver transcriptomes. Approximately 90.73% of these clean reads were mapped to the reference genomes based on a total of 2.32 × 10^6^ species-specific SNPs (Table 1, Additional file 1). The complete clean reads were uploaded to the NCBI Sequence Read Archive (SRA) website (https://www.ncbi.nlm.nih.gov/sra/) under accession number PRJNA679638.

**Table 1:** Basic information on the intestinal and liver transcriptomes of the two dietary groups of fish

Fish group	Total clean reads	Clean reads bases (bp)	SNP Number	Unique mapped reads	Total mapped reads(%)
C-Intestine1	40,875,328	6,120,857,148	178,115	33,355,355 (81.60%)	37,659,356 (92.13%)
C-Intestine2	44,897,254	6,724,235,532	172,520	36,725,040 (81.80%)	41,284,036 (91.95%)
C-Intestine3	45,432,496	6,801,174,866	176,720	36,842,441 (81.09%)	41,430,875 (91.19%)
H-Intestine1	45,623,958	6,831,303,448	469,045	38,470,050 (82.10%)	40,048,325 (87.78%)
H-Intestine2	45,240,496	6,777,495,390	388,932	35,867,617 (82.10%)	40,857,373 (90.31%)
H-Intestine3	45,129,468	6,760,490,246	296,917	32,785,787 (82.43%)	40,738,675 (90.27%)
H-Liver1	40,301,740	6,031,302,492	124,038	35,510,632 (77.83%)	37,363,894 (92.71%)
H-Liver2	50,305,328	7,536,275,016	134,514	36,584,989 (80.87%)	46,520,571 (92.48%)
H-Liver3	42,994,402	6,436,248,490	113,674	36,549,783 (80.99%)	39,787,469 (92.54%)
C-Liver1	46,856,834	7,010,976,494	92,877	33,170,072 (82.30%)	43,343,128 (92.50%)
C-Liver2	43,690,178	6,540,010,734	92,342	41,286,506 (82.07%)	35,867,617 (82.10%)
C-Liver3	39,773,512	5,952,176,384	87,825	35,407,930 (82.35%)	36,940,518 (92.88%)

C-Intestine1 ~ 3 and C-Liver1 ~ 3: three intestinal and liver tissues of hybrid fish from carnivorous diet group, respectively. H-Intestine1 ~ 3 and H-Liver1 ~ 3: three intestine and liver tissues of hybrid fish from herbivorous diet group, respectively

**Fig. 2:**
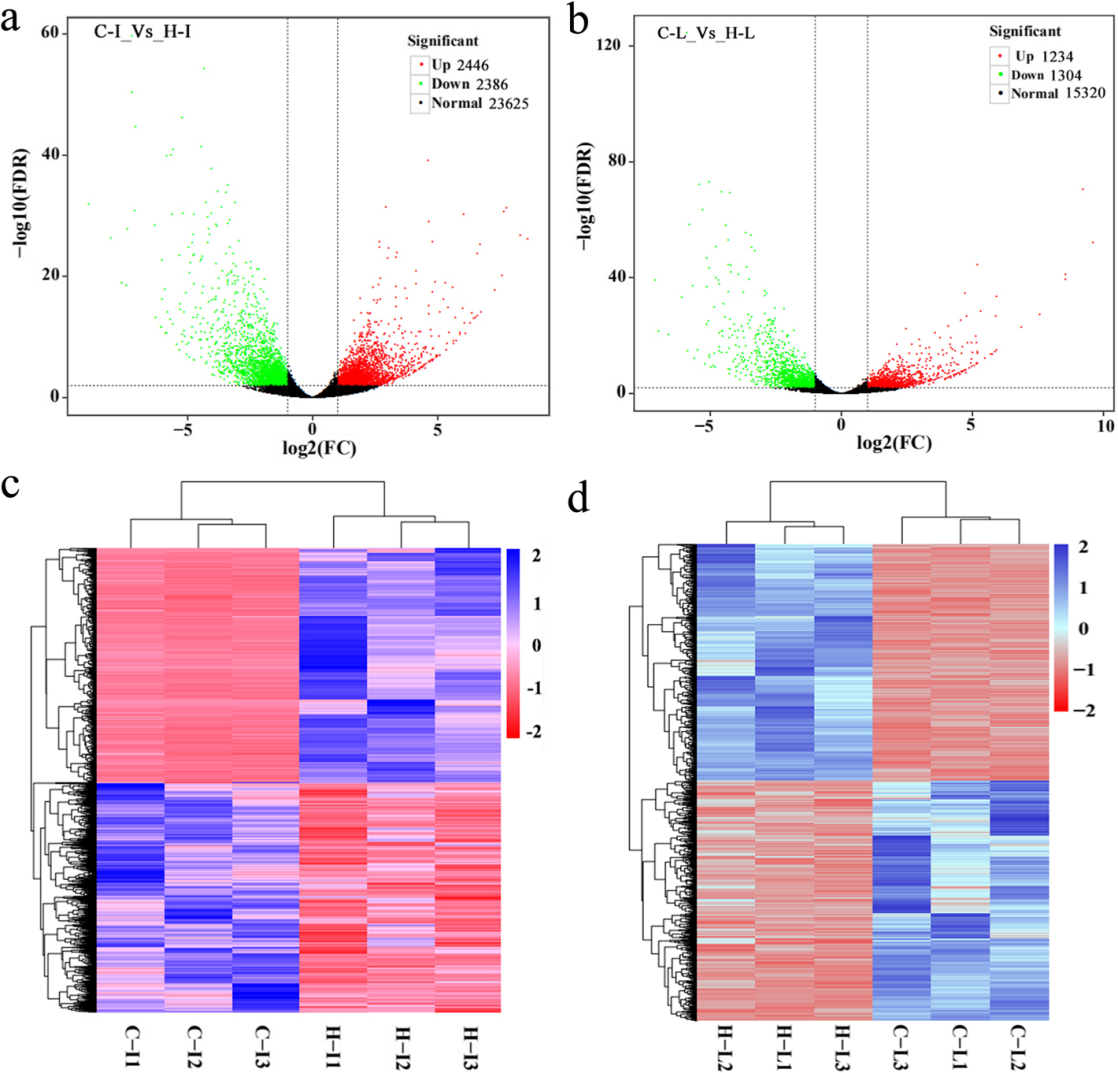
Cluster analysis of differentially expressed homoeologous genes in intestinal **a**, **c** and liver **b**, **d** transcriptomes of different dietary groups of fish. The colors and numbers (log10 fold changes) indicate changes in expression levels. C-I1 ~ I3 and C-L1 ~ L3: intestinal and liver tissues from fish fed carnivorous diet, H-I1 ~ I3 and H-L1 ~ L3: intestinal and liver tissues from fish fed herbivorous diet

Based on the number of mapped reads and genome annotation, DEHs between the two dietary groups of fish were detected (Additional file 2, Table S1). A total of 28,457 homoeologous genes were coexpressed in the intestine. Among these genes, 4832 DEHs were detected, including 2446 that were more highly expressed and 2386 that were less highly expressed in fish fed herbivorous diet than in fish fed carnivorous diet (Fig. 2a, c). A total of 17,858 genes were coexpressed in the liver, and 2538 DEHs were detected, including 1234 that were more highly expressed and 1304 that were less highly expressed in fish fed with herbivorous diet than in fish fed with carnivorous diet (Fig. 2b, d). The top 24 DEHs in the intestine and liver tissues are presented in Table 2. Compared with fish fed carnivorous diet, some DEHs, such as *sqlea*, *hmgcra*, *pck1*, *ulk2* and *dio3b*, were detected in both intestine and liver tissues in fish fed herbivorous diet.

**Table 2:** Top 24 differentially expressed homoeologous genes in the intestinal and liver tissues from herbivorous diet group compared with carnivorous diet group

DEHs in intestine			DEHs in liver		
Gene symbol	Log2 (fold change)	KO ID	Gene symbol	Log2 (fold change)	KO ID
*cyp7a1*	8.30	K00489	*sqlea*	9.59	K00511
*cxcl8b.3*	7.76	K10030	*hmgcra*	9.22	K00021
*sqlea*	6.75	K00511	*cyp51*	5.91	K05917
*col12a1b*	5.64	K08132	*mcm6*	5.87	K02542
*fgf4*	5.59	K04358	*ntd5*	5.76	K17305
*rpe65b*	5.40	K20991	*am2*	5.47	K03910
*clca5.2*	4.98	K05030	*mcm2*	5.20	K02540
*lamc3*	4.95	K06247	*epoa*	5.20	K05437
*tubb5*	4.89	K07375	*hells*	5.17	K19001
*ppp1r3ca*	4.79	K07189	*hsd17b7*	5.16	K13373
*fuk*	4.76	K05305	*mcm3*	5.16	K02541
*hmgcra*	4.73	K13187	*ebp*	5.12	K01824
*mat1a*	−7.13	K00789	*klf13*	−5.91	K09208
*diabloa*	−7.09	K10522	*pck1*	−5.80	K01596
*pck1*	−6.01	K01596	*lpin1*	−5.30	K15728
*ulk2*	−5.84	K08269	*lipg*	−5.15	K01046
*slc6a19a*	−5.79	K05334	*foxo1a*	−5.05	K07201
*slc26a6*	−5.71	K14704	*arg2*	−5.02	K01476
*bco1*	−5.28	K00515	*slc25a48*	−4.77	K15124
*dio3b*	−5.19	K07754	*tdo2*	−4.69	K00453
*slc15a1b*	−4.97	K14206	*pdzrn3a*	−4.66	K15682
*pde9al*	−4.63	K13761	*ulk2*	−4.66	K08269
*ucp1*	−4.58	K15103	*sik2b*	−4.65	K16311
*ctrl*	−4.50	K09632	*dio3b*	−4.53	K07754

Specifically expressed genes were detected among different tissues and diets. Seventeen and 11 homoeologous genes were specifically expressed in the liver and intestinal of fish fed herbivorous diet, respectively, and none were specifically expressed in fish fed carnivorous diet (Table S2). In addition, 569 homoeologous genes were specifically expressed in the intestine, and 48 were specifically expressed in the liver, showing no correlation with dietary adaptation (Additional file 3). One homoeologous gene, Rho GTPase-activating protein (*arhgap29*), was detected in the intestine and liver in only the fish fed herbivorous diet (Additional file 3).

KEGG functional cluster analysis revealed that the intestinal DEHs were enriched in 364 pathways and that the liver DEHs were enriched in 345 pathways. Those DEHs were highly enriched in PPAR signaling pathway (ko03320); biosynthesis of secondary metabolites (ko01110); glycine, serine and threonine metabolism (ko00260); cysteine and methionine metabolism (ko00270); glycerolipid metabolism (ko00561); fat digestion and absorption (ko04975) associated with metabolism in the two dietary groups of fish. The top 15 enriched pathways associated with metabolism in the two dietary groups are presented in Table 3. Interestingly, genes involved in C5 isoprenoid biosynthesis and mevalonate pathway (Fig. S1) were significantly more highly expressed, and genes involved in the insulin signaling pathway (Fig. S2) were significantly less highly expressed, in both the liver and intestine of fish fed herbivorous diet than those in fish fed carnivorous diet.

**Table 3:** Top 15 enriched pathways associated with differentially expressed homoeologous genes related to metabolism in herbivorous diet group compared with carnivorous diet group

**Pathway in intestine transcriptome**	**Gene NO.**	**Reference NO.**	***P*** **value**	**Pathway ID**
Biosynthesis of antibiotics	109	459	1.43E-13	ko01130
PPAR signaling pathway	40	134	1.13E-08	ko03320
Carbon metabolism	59	240	1.60E-08	ko01200
Steroid biosynthesis	19	40	2.02E-08	ko00100
Fat digestion and absorption	27	75	3.65E-08	ko04975
Biosynthesis of secondary metabolites	138	757	4.06E-08	ko01110
Cholesterol metabolism	33	111	2.36E-07	ko04979
Glycine, serine and threonine metabolism	28	86	2.39E-07	ko00260
Cysteine and methionine metabolism	28	87	3.14E-07	ko00270
Biosynthesis of amino acids	42	165	6.80E-07	ko01230
Fatty acid biosynthesis	15	34	2.06E-06	ko00061
Proximal tubule bicarbonate reclamation	19	53	4.09E-06	ko04964
Protein digestion and absorption	53	244	5.08E-06	ko04974
Glycolysis / Gluconeogenesis	32	129	2.46E-05	ko00010
Citrate cycle (TCA cycle)	19	62	5.23E-05	ko00020
**Pathway in liver transcriptome**	**Gene NO.**	**Reference NO.**	***P*** **value**	**Pathway ID**
Biosynthesis of antibiotics	80	459	2.85E-15	ko01130
Metabolic pathways	257	2479	8.47E-14	ko01100
Biosynthesis of secondary metabolites	103	757	4.59E-12	ko01110
Steroid biosynthesis	16	40	2.40E-09	ko00100
Glycine, serine and threonine metabolism	22	86	4.08E-08	ko00260
FoxO signaling pathway	47	303	7.83E-08	ko04068
Insulin signaling pathway	49	323	8.44E-08	ko04910
Cysteine and methionine metabolism	21	87	2.42E-07	ko00270
PPAR signaling pathway	27	134	2.68E-07	ko03320
Cholesterol metabolism	24	111	3.14E-07	ko04979
AMPK signaling pathway	44	298	8.28E-07	ko04152
Terpenoid backbone biosynthesis	14	46	1.24E-06	ko00900
Glyoxylate and dicarboxylate metabolism	17	68	1.99E-06	ko00630
Carbon metabolism	35	240	1.43E-05	ko01200
Adipocytokine signaling pathway	27	169	2.64E-05	ko04920

### Homoeolog expression silencing and bias associated with dietary adaptation

**Fig. 3:**
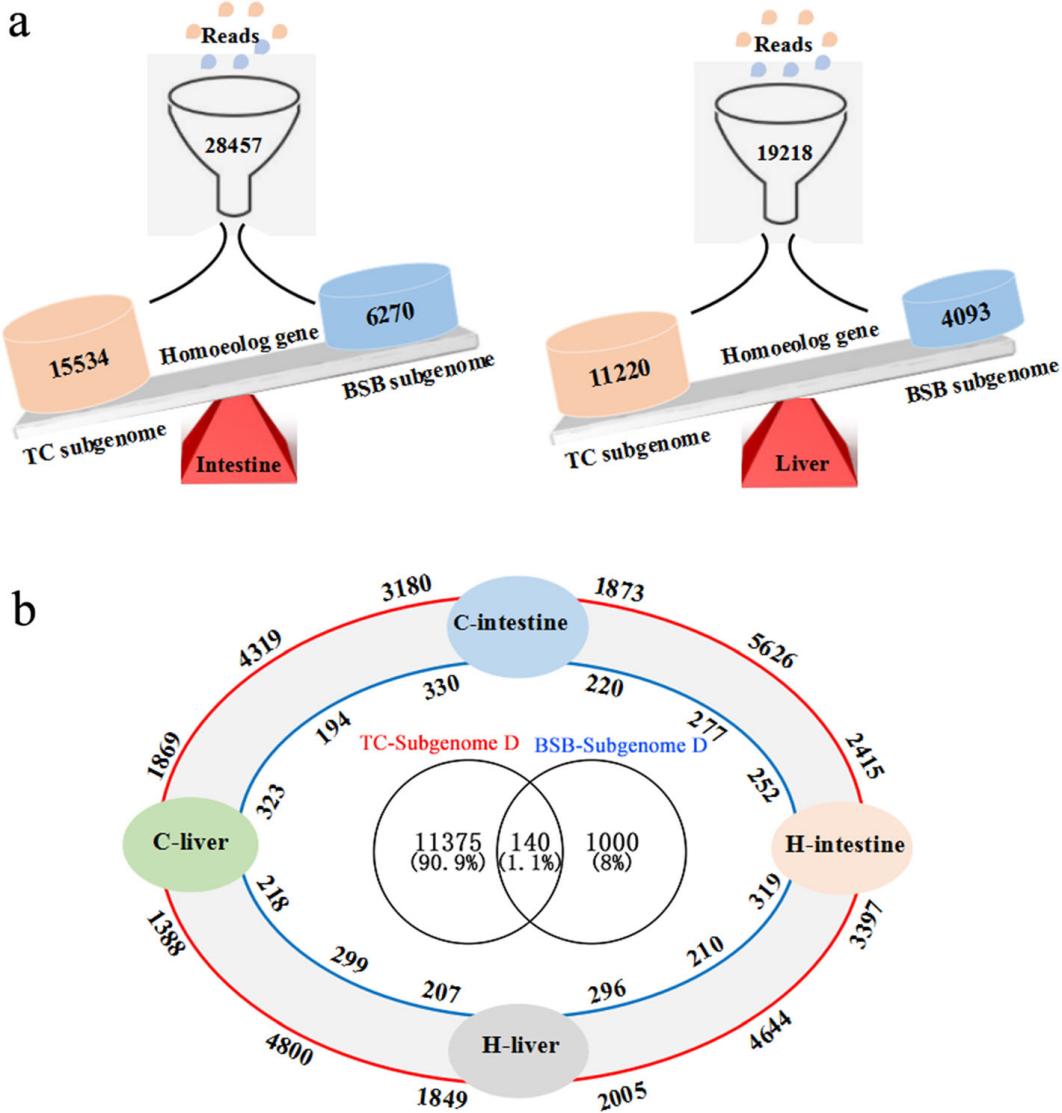
Subgenome expression dominance in intestinal and liver tissues **a** and the shared and specially expressed homoeologous genes in different tissues **b**. The number on the red and blue lines represent the shared and specifically expressed homoeologous genes with TC and BSB subgenome expression dominance, respectively

Based on the mapped read data, subgenome dominance were observed in all tissues, with homoeologous genes derived from parent TC being significantly more highly expressed than those derived from parent BSB. For example, a total of 28,457 homoeolog genes were coexpressed in intestinal tissue, including 6270 annotated to BSB genome, 15,534 annotated to TC genome and 6653 newly assembled genes. In liver tissues, a total of 17,858 genes were coexpressed, including 3312 annotated to BSB genome, 10,699 annotated to TC genome and 3847 newly assembled genes (Fig. 3a, Additional file 2).

Analysis of homoeolog expression silencing (HES) and homoeolog expression bias (HEB) indicated that the TC subgenome was dominant in all tissues (Additional file 4). The amount of BSB-HES was significantly greater than the amount of TC-HES in all tissues, while the amount of TC-HEB was significantly greater than that BSB-HEB (*p* < 0.05), indicating that the homoeologous genes derived from the parent were unbalanced in the hybrid fish during dietary adaptation (Table 4). Besides, analysis of the shared and specifically expressed homoeologous genes indicated that subgenome dominance was correlated with tissues and almost completely unrelated to diet (Fig. 3b). For instance, the number of homoeologous genes that showed BSB subgenome dominance in the liver and intestine was greater than the number of specially expressed homoeolog genes. The number of homoeolog genes that showed TC subgenome dominance was greater than the number of specifically expressed genes in all tissues (Fig. 3b).

**Table 4:** Homoeolog genes expression silencing and bias in tissues from the two dietary groups of fish

Type	TC subgenome dominance		BSB subgenome dominance		Normal	Total
	BSB-HES	TC-HEB	TC-HES	BSB-HEB		
C-Intestine	4522 (44.92%)	2977 (29.57%)	243 (2.41%)	254 (2.52%)	2070 (20.56%)	10,066
H-Intestine	4132 (37.17%)	3909 (35.17%)	233 (2.10%)	296 (2.66%)	2596 (23.35%)	11,166
C-Liver	4482 (54.95%)	1706 (20.91%)	319 (3.91%)	198 (2.43%)	1452 (17.80%)	8157
H-Liver	4334 (49.32%)	2315 (26.34%)	309 (3.52%)	197 (2.24%)	1633 (18.58%)	8788

**Fig. 4:**
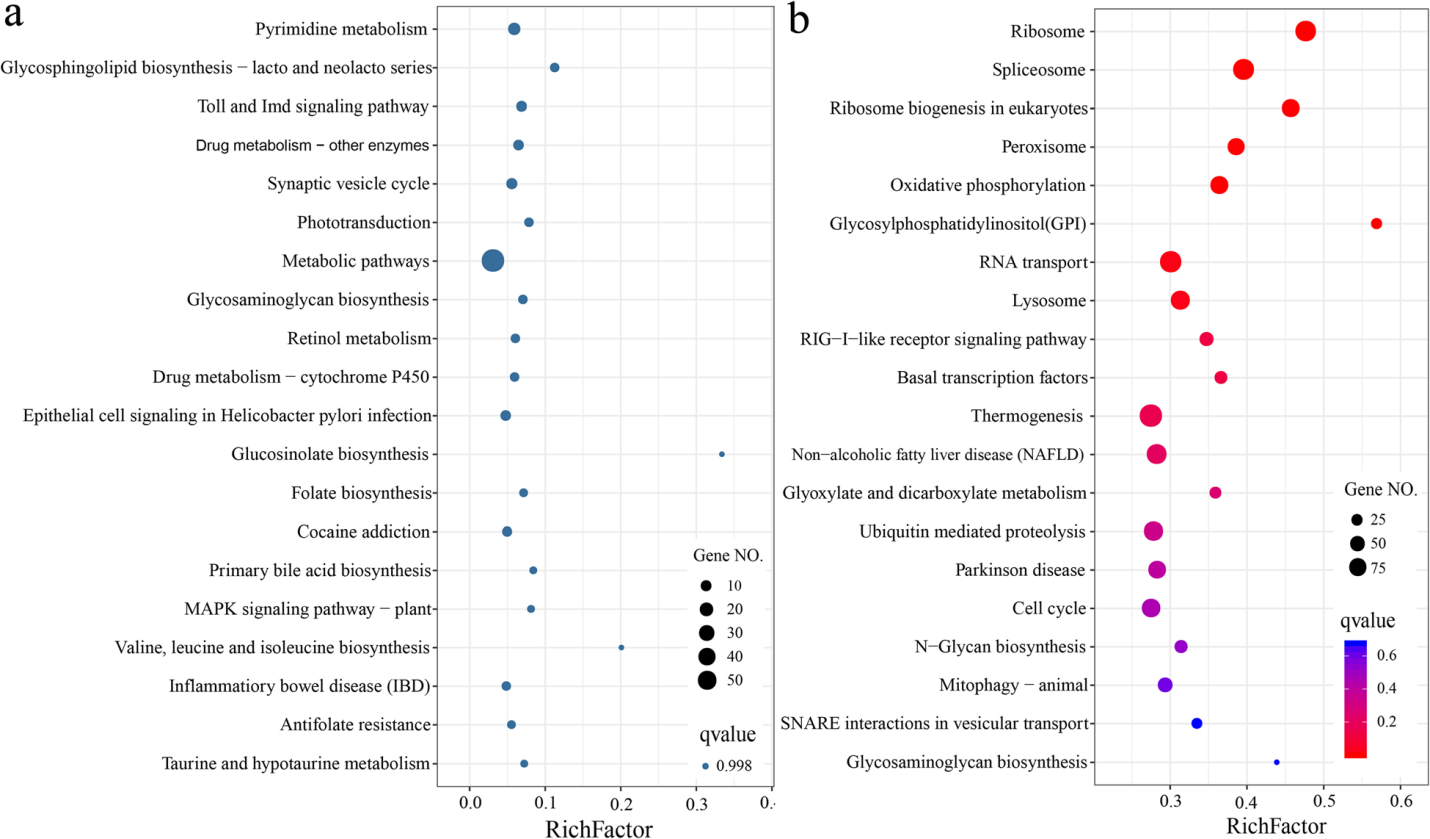
Top 20 enriched pathways of the expressed homoeologous genes with BSB subgenome expression dominance **a** and TC subgenome expression dominance **b** in the two dietary groups of fish

**Fig. 5:**
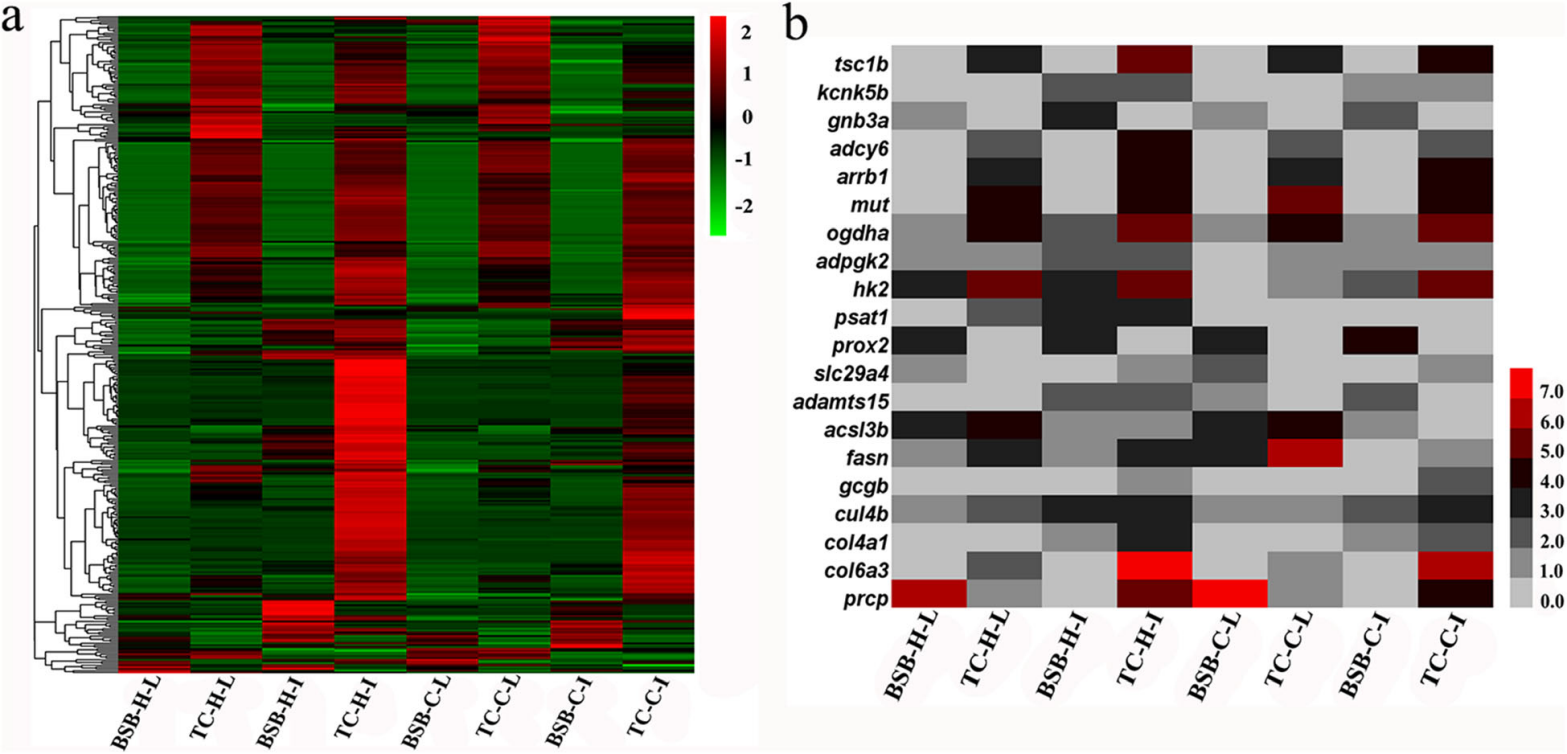
Relative expression levels of the coexpressed homoeologous genes associated with food intake, digestion and metabolism in the two dietary groups of fish. Global expression level of the coexpressed homoeologous genes involved in taste transduction, olfactory transduction, carbon metabolism, starch and sucrose metabolism, fatty acid biosynthesis and protein digestion and absorption pathways **a**. Homoeologous genes that showed subgenome expression dominance **b**. The colors and numbers (log (fold change+ 1, 2)) indicated changes in expression levels. H-L and C-L: represent liver transcriptomes in fish from herbivorous and carnivorous diet groups; H-I and C-I: represent intestinal transcriptomes in fish from herbivorous and carnivorous diet groups

COG functional analysis revealed that the homoeologous genes that showed subgenome dominance were highly enriched in translation ribosomal structure and biogenesis, posttranslational modification, protein turnover, chaperones, carbohydrate transport and metabolism and signal transduction mechanisms (Additional file 5). KEGG functional enrichment analysis revealed that the parent subgenome controlled certain pathways during dietary adaptation. For instance, BSB subgenome mainly controlled metabolism and diseases pathways, such as pyrimidine metabolism, glycosphingolipid biosynthesis, glycosaminoglycan biosynthesis, retinol metabolism, glucosinolate biosynthesis, epithelial cell signaling in *Helicobacter pylori* infection, and antifolate resistance. The TC subgenome mainly controlled genetic information processing and cellular processes pathways, such as ribosome, spliceosom, aminoacyl-tRNA biosynthesis, basal transcription factors, ubiquitin mediated proteolysis, peroxisome, lysosome, cell cycle, and p53 signaling pathway (Fig. 4, Additional file 5). The relative expression levels of the homoeologous genes associated with food intake, digestion and metabolism, including olfactory transduction, taste transduction, carbon metabolism, starch and sucrose metabolism, fatty acid biosynthesis and protein digestion and absorption are presented in Fig. 5.

### qPCR validation

**Fig. 6:**
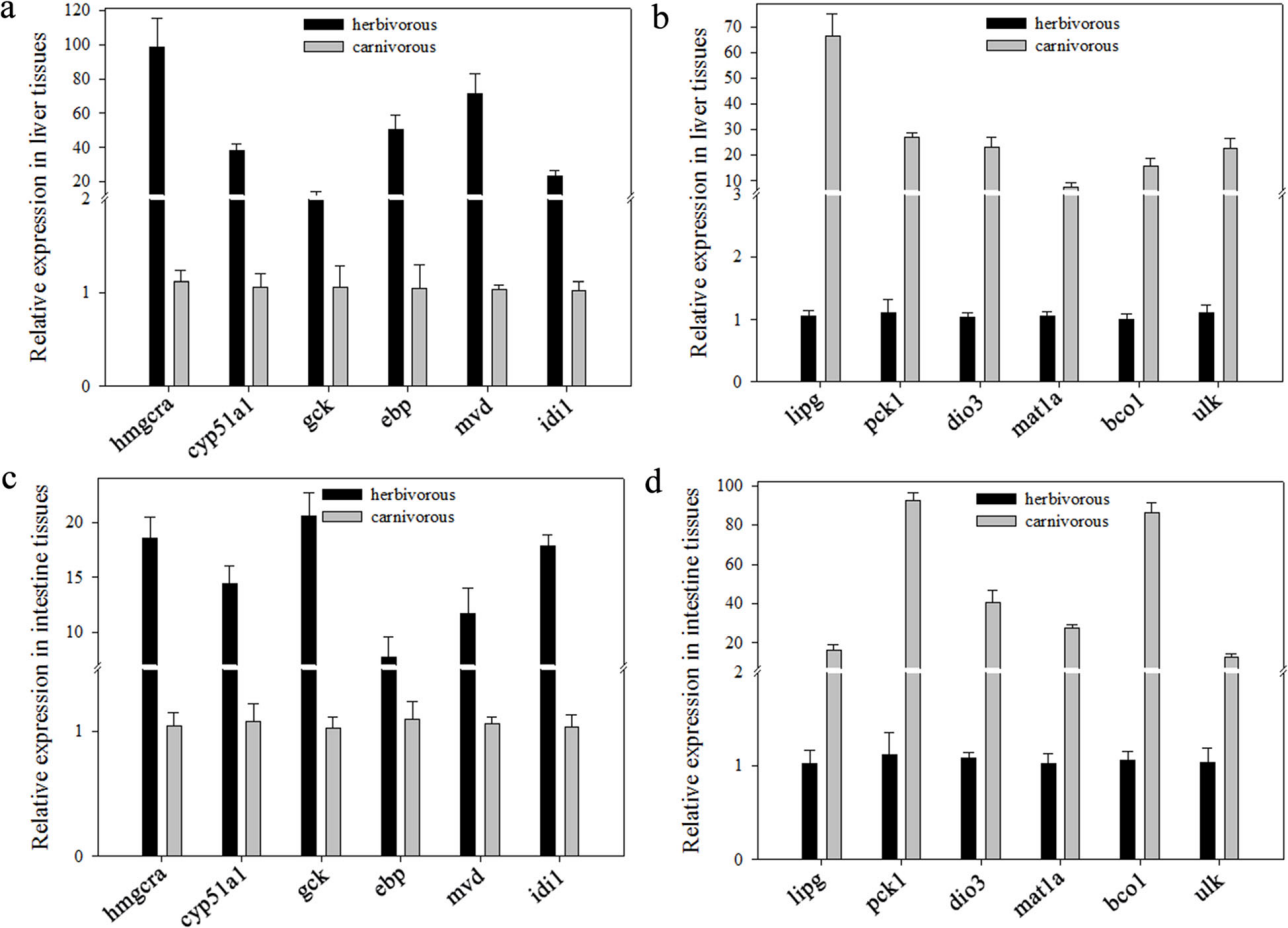
Real-time polymerase chain reaction analysis of 12 differentially expressed genes between the two dietary groups of fish. Higher and lower expression levels of homoeologous genes in the liver **a** and **c** and intestine **b** and **d** of fish fed herbivorous diet compared with fish fed carnivorous diet, respectively. The genes included 3-hydroxy-3-methylglutaryl-CoA reductase (*hmgcra*), cytochrome P450 family 51 subfamily A member 1 (*cyp51a1*), glucokinase (*gck*), EBP cholestenol delta-isomerase (*ebp*), mevalonate diphosphate decarboxylase (*mvd*), isopentenyl-diphosphate delta isomerase 1 (*idi1*), lipase G, endothelial type (*lipg*), phosphoenolpyruvate carboxykinase 1 (*pck1*), iodothyronine deiodinase 3 (*dio3*), methionine adenosyltransferase 1A (*mat1a*), beta-carotene oxygenase 1 (*bco1*) and Unc-51 like autophagy activating kinase 1 (*ulk1*)

To validate the quality of the RNA-seq data, we randomly chose 12 DEHs involved in mevalonate pathway and fatty acid biosynthesis pathway (including 3-hydroxy-3-methylglutaryl-CoA reductase (*hmgcra*); glucokinase (*gck*); EBP cholestenol delta-isomerase (*ebp*); isopentenyl-diphosphate delta isomerase 1 (*idi1*); bytochrome P450 family 51 subfamily A member 1 (*cyp51a1*), mevalonate diphosphate decarboxylase (*mvd*); methionine adenosyltransferase 1A (*mat1a*); lipase G, endothelial type (*lipg*); phosphoenolpyruvate carboxykinase 1 (*pck1*); Unc-51-like autophagy activating kinase 1 (*ulk1*); iodothyronine deiodinase 3 (*dio3*); beta-carotene oxygenase 1 (*bco1*)) and performed qPCR with three biological replicates. The relative expression levels of the 12 DEHs are presented in Fig. 6. The trends of the expression levels of these genes detected by qPCR were the same as those obtained by RNA-seq data analysis. These results indicated the reliability of the RNA-seq data for the analysis of differentially expressed homoeoloousg genes during diet adaptation.

### Liver biochemical assays

The concentrations of GSH, MDA, T-SOD and POD in the liver tissue from carnivorous and herbivorous diet fish are presented in Table 5. No significant differences were observed in the GSH, T-SOD and POD between the two dietary groups of fish. However, the concentrations of MDA were significantly higher in fish fed with carnivorous diet than in fish fed with herbivorous diet (*p* < 0.05, *t-test*).

**Table 5:** Biochemical assays of liver tissues in the two dietary groups of fish

Type	GSH (g/L)	MDA (nmol/mL)	T-SOD (U/mL)	POD (U/mL)
Carnivorous diet	50.63 ± 2.46	182.21 ± 16.51*	39.23 ± 0.49	27.18 ± 0.67
Herbivorous diet	59.03 ± 2.63	4.02 ± 0.33	41.73 ± 0.44	23.93 ± 0.39

* *p* < 0.05, representing significant difference between the two groups

## Discussions

### Diet shifts and adaption affect phenotypic traits

Fish can change their behavioral and physiological characteristics, such as food selection, food intake, enzyme production, and alimentary tract development, to adjust to changes in the quality of available food [27, 29]. Generally, these adaptive changes are limited by the range of phenotypic plasticity in the short term, and long-term adaptability depends on the evolution of genotype. Previous studies reported that the differences of histological structures among fish gastrointestinal tracts are related to feeding habits, food, age and weight [30, 31]. In this study, the allodiploid hybrid fish adapted to carnivorous and herbivorous diets also showed a significant difference in histological structures of liver and intestinal tissues (Fig. 1), indicating that food type is associated with the developmental process of gastrointestinal tracts [14, 15].

Genetic adaptation to dietary environments is a key process in the evolution of natural populations and is of great interest in fish breeding, as the ability to adaptation to major diet changes can be effectively used to promote fish welfare and a more sustainable aquaculture. Recent studies have focus more on the effect of feeding changes (substituted diets) on growth performance, metabolism, nutrition and gene regulation of fishes [32–34]. However, dietary changes and adaptation affect the function of hepatic metabolism to varying degrees, which may result in metabolic and other problems, such as reduced antioxidant capacity, disordered glucose and lipid metabolism, and weakened disease resistance [35–37]. In this study, due to the merging of two subgenome from the carnivorous TC and herbivorous BSB, the hybrid fish seemed to have the potential to adapt to carnivorous or herbivorous diets, and in fact, no impact on growth level was observed in the two dietary groups of fish. In addition, the metabolites related to antioxidant protection, such as GSH, T-SOD and POD, in the liver between the two dietary groups of fish were not significantly different. Higher concentrations of MDA were detected in the carnivorous diet group than in the herbivorous diet group (Table 5), indicating that adaptation to the carnivorous diet more than that to the herbivorous diet affects hepatic metabolism in the hybrid fish. In addition, the changes in metabolic enzyme activity need to be further studied.

### Diet shifts and adaption affect metabolic pathways

Diet shifts and long-term adaptation can affect major nutrient (carbohydrate, protein and lipid) digestion and metabolism and often lead to significant metabolic changes, such as the modification of certain key metabolic enzymes [35, 38, 39], hepatic metabolic pathways, including energy metabolism, cholesterol biosynthesis and proteolytic activity [35, 38, 40, 41]. Here, we identified a number of DEHs in the hybrid fish adapted to herbivorous diets and carnivorous diets (Fig. 2 and Table 2). These DEHs are highly enriched in carbon metabolism, steroid biosynthesis and amino acid, fatty and protein metabolism (Table 3, Fig. S2 and S3), indicating that diet derives genetic adaption and adjustment to the available food. On the other hand, metabolic adaption of these pathways apparently supports hybrid fish adaptation to carnivorous and herbivorous diets. In European sea bass (*Dicentrarchus labrax*), genes involved in the LC-PUFA and cholesterol biosynthetic pathways also upregulated when the fish were fed with plant-based diet compared with fish-based diet [42]. In grass carp (*Ctenopharyngodon idellus*), an herbivorous fish, genes in steroid biosynthesis, terpenoid backbone biosynthesis and glycerophospholipid metabolism pathways were differetinally expressed during carnivorous transition to an herbivorous diet [43]. In BSB, 8 weeks of feeding with a high-fat carbohydrate diet also caused a significant decline in the numbers of amino acids entering TCA cycle, which in turn resulted in elevated levels of seven amino acids [44].

Insulin serves as the primary regulator of blood glucose balance, regulating the metabolism and storage of nutrients such as protein, sugar and fat by acting on liver, muscle and adipose tissue [45]. In BSB, 8 weeks of feeding with a high-carbohydrate diet upregulated the genes associated with insulin signaling pathways, which may lead to the development of insulin resistance in hepatocytes, pathological liver changes, and, eventually, nonalcoholic fatty liver disease [35]. Our study also identified that the insulin receptor, insulin receptor-related receptor and downstream pathways, including PI (3) K, Akt, FOXO1, Cbl protooncogene and mTOR, were expressed at lower levels in the fish that fed herbivorous diet (Fig. S2), suggesting that the adaptation of hybrid fish to a carnivorous diet may also caused insulin imbalance and insulin resistance in hepatocytes [35, 45].

### Asymmetric expression of homoeologous genes contributes to dietary adaptation

The merged subgenomes in a polyploid must adjust and coexist with one another in a single nucleus, which may cause interactions between divergent regulatory networks due to differential dosage sensitivity and epigenetic alterations. The variation in the expression of the homoeologous gene is easily observed. To some extent, investigating the variation in the expression of homeolog genes is hindered by at least two major limitations: (1) lack of genomic resources for extant parental progenitors and (2) the inability to distinguish the origins of the subgenome. In our previous study, we completed whole-genome sequencing of the two parents BSB and TC [28], which provided basic date for the present study. Here, subgenome dominance were observed in different tissues, and paternal TC subgenome, more than maternal BSB subgenome dominant expressed during dietary adaptation (Fig. 3). One possible outcome is that the newly formed hybrid must resolve genetic incompatibilities very rapidly within the first generation to adapt environment, including the diet selection and adaptation [7, 9]. Besides, subgenome expression dominance may be associated with inherent dominance between subgenomes. We also detected the silenced homoeologous genes was unbalanced in tissues, and the silenced homoeologous genes was significantly greater than the bias homoeologous genes during dietary adaptation (Table 4). This may be associated with various regulation elements, such as cis- and trans-regulatory element, low-transposability elements and DNA methylation [46, 47]. However, the underlying mechanisms for preferential silencing of one parental subgenome over other subgenomes and their functions remain to be further studied.

How one subgenome becomes more highly expressed and whether the direction of subgenome dominance is external induced in a polyploid remain largely unknown. However, a possible scenario in which genetic incompatibilities are resolved and subgenome expression dominance is achieved is when certain subsets of pathways are controlled by one subgenome, while another subgenome controls the remaining set of pathways [11, 48]. Obviously, this would result in the partitioning of phenotypic traits to different subgenomes and is of great significance in genetic breeding of plants and animals. Subgenome dominance contributing to heterosis, such as phenotypic bias, has been observed in several allopolyploid plants [9–12]. In the present study, we also detected parent subgenome controlled functional pathways during dietary adaptation. The BSB subgenome mainly controlled metabolic and diseases pathways, while the TC subgenome mainly controlled genetic information processing and cellular processes (Fig. 4). These pathways, such as the circadian rhythm, digestion and metabolism pathway, were also activated in grass carp during the transition from a carnivorous diet to an herbivorous diet [43, 49]. Altered expression of pathways involved in retinal photosensitivity, circadian rhythm and appetite control were observed in mandarin fish (*Sinipercidae*) feed on only live prey fish [50]. Thus, our results indicated that subgenome dominance may play crucial roles in the adaptation of hybrid fish to herbivorous and carnivorous diets. In addition, it would be interesting to investigate whether subgenome expression dominance is heritable and contributes to diet selection in F_2_-F_5_ of this hybrid lineage.

## Conclusions

In summary, the hybrid fish derived from herbivorous BSB (♀) × carnivorous TC (♂) exhibited altered histological structures of liver and intestinal tissues during adaptation to herbivorous and carnivorous diets. Furthermore, homoeologous genes involved metabolism pathways, such as fat, carbon, protein and amino acid metabolism, were differentially expressed during the two types of dietary adaptation. Moreover, subgenome dominance was observed in the hybrid fish, and the direction of expression dominance almost irrelevant to dietary adaptation process. In addition, subgenome expression dominance controlled functional pathways during adaptation to herbivorous and carnivorous diets. These results indicated that diet could drive phenotypic and genetic variation, and subgenome expression dominance may play key roles in dietary adaptation in the hybrid fish. This study can provide basic data for the investigation of the signal transduction pathways that mediate food digestion, metabolism and adaption in fish and also may provide perspectives for fish breeding and application.

## Methods

### Experimental fish and sampling

Broodstock BSB and TC were originally obtained from Dong Ting Lake (Hunan province, China). During the reproduction season, from May to June in 2019, sexually matured BSB (female = 5) and TC (male = 5) were choose as two parents and fish crossing were performed as described previously [26]. Approximately 400 larvae from female BSB × male TC were randomly chosen and reared in two 200-L aquaria. During the breeding process, water temperature was controlled at 25.5 ± 0.5 °C under natural photoperiod. The aquaria contained circulating water system, and between 20 and 30% of the water was replaced every 3 days. The experimental fish fed with *Artemia* routinely three times a day until 50 days of age. Then, 180 individuals were randomly collected and divided into two group. Each group fish were reared in three 200-L aquaria (each *n* = 30). One group fish fed *Chironomid larvae* (defined as carnivorous) and the other fed *duckweed* and artificial fodder (defined as herbivorous) routinely two times a day. The artificial fodder included the following components (per 100 g): fish meal 5.00 g, soybean meal 30.00 g, rapeseed meal 20.00 g, rice bran 35.00 g, fish oil 3.50 g, among others. The amount of food source was gradually increased according to the fish’s body weight. The two types of fish (each = 6) were sampled at 120 days of age after a 12-h fast. The body weights were 21.75 ± 1.49 g and 20.11 ± 0.96 g for fish fed the carnivorous and herbivorous diet, respectively, and the folk length were 11.63 ± 0.46 cm and 11.51 ± 0.55 cm. Liver and intestinal tissues were removed, washed with DEPC water and immediately placed into RNALater (Ambion Life Technologies, USA) for mRNA analysis. A piece of liver and intestinal tissue was placed into Bouins’ fixative for histological analysis, and the remaining liver tissue was collected and stored in − 70 °C freezer for biochemical assays.

### RNA extraction, library, sequencing and mapping

Total RNA from intestine tissues and liver were extracted using TRIzol® Reagent, Invitrogen™ (Life Technologies) following the manufacturer’s instructions. After RNA quality assessment, 1.0 μg of total RNA per sample was used as input material for the RNA sample preparations. Sequencing libraries were generated using NEBNext UltraTM RNA Library Prep Kit for Illumina (NEB, USA). First-strand cDNA was synthesized using random hexamer primers and M-MuLV reverse transcriptase. Second-strand cDNA synthesis was subsequently performed using DNA polymerase I and RNase H. After adenylation of 3′ ends of DNA fragments, NEBNext adaptors with hairpin loop structures were ligated to prepare for hybridization. The library fragments were purified with AMPure XP system (Beckman Coulter, Beverly, USA) to preferentially select cDNA fragments that were 240 bp in length. Then, 3.0 μl of USER Enzyme (NEB, USA) was used with size-selected, adaptor-ligated cDNA at 37 °C for 15 min followed by 5 min at 95 °C before PCR. Next, PCR was performed with Phusion High-Fidelity DNA polymerase, universal PCR primers and index (X) Primer. Finally, PCR products were purified and sequenced on an Illumina HiSeq 2000 platform.

In this study, the genomes of parents BSB (30,749 annotated genes) and TC (31,022 annotated genes) from our previous study were used as two reference genomes [28]. After obtaining the paired-end raw reads of each sample, FastQC software (Babraham Bioinformatics) was used to remove reads containing adapter, ploy-N and low quality sequences (unknown nucleotides > 5%) [51]. Then, the total clean reads of each sample were aligned to the two reference genomes by using HISAT software based on the species-specific SNPs [52]. The number of mapped reads in each gene was calculated with some in-house Perl scripts as previously described [28]. Only reads with a perfect match or one mismatch were further analyzed and annotated based on the two reference genomes. Furthermore, to obtain more comprehensive annotation information, StringTie software was used to assemble the mapped reads [52]. After removing the short sequences that encoded peptide chain less than 50 amino acid or contains only a single exon, a total of 11,282 new genes were generated, and 9606 can be annotated to at least one databases, including Nr, Nt, Pfam, KOG/COG, Swiss-Prot, KO and GO terms.

### Differential expression analysis

Quantification of gene expression levels was estimated by fragments per kilobase of transcript per million fragments mapped (FPKM) method based on the mapped reads [53]. The expression values of three biological replicates were screened with a mean ± 2 standard deviation (SD) threshold in each gene to avoid interference from expression noise [54]. In addition, the total expression value was normalized based on the ratio of the number of mapped reads for each gene to the total number of mapped reads for the entire genome. Finally, DEseq2 in R software, version 2.13 (R Foundation for Statistical Computing, Vienna, Austria), was used to search for differentially expressed homoeologous genes (DEHs) between the two dietary groups of fish with a false discovery rate (FDR) < 0.01 and a threshold normalized absolute log 2-fold change > 1.0 [55]. We also detected the special expressed homoeologous genes between the two dietary groups of fish and different tissues. Specially expressed genes were defined as genes with an expression level of zero (read count = 0 in three biological replicates) in one tissues or one dietary group of fish (while in the other group or tissues: the read count was ≧ 3 in three biological replicates). Gene Ontology (GO) and KEGG pathway analyses were carried out on the free cloud platform BMKCloud (https://international.biocloud.net/zh/user/login) based on the BSB and TC genome annotation.

### Analysis of homoeolog expression silencing and bias

Two sets of genome sequences (annotated protein sequences) were aligned using the reciprocal BLAST (BLASTP, v. 2.2.26) hit method with an e-value cut off of 1e-^20^ to identify orthologs. A total of 20,130 orthologs were obtained for subsequent analysis. Then, the mapped reads of each gene were divided into two categories based on the two different parental reference genomes. BSB homoeolog-specific reads (BSB)/TC homoeolog-specific reads (TC) were used to detect homoeologs expression silencing (HES) and expression bias (HEB), respectively [56]. Expressed genes were defined as BSB silenced (BSB-HES) if BSB = 0 and TC ≧ 3 and TC silenced (TC-HES) if BSB ≧ 3 and TC = 0 in the three biological replicates. For analysis of HEB, the expressed genes with BSB = 0 and TC ≦ 3 or BSB ≦ 3 and TC = 0 were first removed. Then, the expression level of gene was considered biased to BSB subgenome (BSB-HEB) if the ratio of BSB/TC ≧ 2 and biased to TC subgenome (TC-HEB) if the ratio of BSB/TC ≦ 0.5. All others cases were considered “normal”.

### Real-time quantitative PCR (qPCR)

Total RNA was isolated from liver tissues of the two dietary groups of fish using TRIzol reagent (Invitrogen) according to the manufacturer’s instructions. First-strand cDNA was synthesized using a PrimeScript RT Reagent Kit (RR047A, TAKARA) with PrimeScript RT Enzyme at 37 °C for 15 min and at 85 °C for 5 s after genomic DNA removal with DNA eraser. qPCR was performed on triplicate technical replicates and ACTN gene (accession no. GU471241) were used as the internal control for normalization of gene expression. qPCR was performed on LightCycler® 96 (Roche, Switzerland) and the amplification conditions were as follows: 50 °C for 5 min, 95 °C for 10 min, and 40 cycles at 95 °C for 15 s and 60 °C for 60 s. Then, relative quantification was performed, and melting curve analysis was used to verify the generation of a single product at the end of the assay. The average threshold cycle (Ct) was calculated for each sample using the 2^-∆∆Ct^ method. The sequences of the primers used are given in Table S3.

### Biochemical assays

Equal amounts of liver tissue from the two dietary groups fish were collected, and homogenates were used to determine the concentrations of superoxide dismutase (SOD), glutataione (GSH), malondialdehyde (MDA) and peroxidase (POD). The assay kits for SOD (A001–1), GSH (A006–1), MDA (A003–1) and POD (A048–2) were purchased from Nanjingjiancheng Bioengineering Institute (Jiangsu, China), and the experimental protocols followed the manufacturer’s instructions. The data are presented as the mean ± standard deviation (SD), and SPSS Statistics 19.0 (IBM Corp., NY, USA) was used to analyze the significance of differences between groups (unpaired two-tailed analysis, *t test*). The level of statistical significance was set at *p* < 0.05.

## Supplementary Information

**Additional file 1.**

**Additional file 2.**

**Additional file 3.**

**Additional file 4.**

**Additional file 5.**

**Additional file 6: Figure S1.** Steroid biosynthesis pathway and the differentilly expressed homoeologous genes in the herbivorous diet group compared with carnivorous diet group. Genes in red boxes were upregulated, and those in green boxes were downregulated.

**Additional file 7: Figure S2.** Insulin signaling pathway and the differentilly expressed homoeologous genes in the herbivorous diet group compared with carnivorous diet group. Genes in red boxes were upregulated, and those in green boxes were downregulated.

**Additional file 8.** Supplement tables (Table S1, Table S2 and Table S3).

## Data Availability

The complete clean reads were uploaded to the NCBI Sequence Read Archive (SRA) website (https://www.ncbi.nlm.nih.gov/sra/) under accession number PRJNA679638. All data generated or analysed during this study are included in this published article and its supplementary information files.

## Abbreviations

BSB: *Megalobrama amblycephala*
TC: *Culter alburnus*
DEHs: Differentially expression homoeologous genes
HES: Homoeolog expression silence
HEB: Homoeolog expression bias.
KEGG: Kyoto Encyclopedia of Genes and Genomes
KO: KEGG orthology
GO: Gene ontology
COG: Cluster of Orthologous Groups of proteins
qPCR: Quantitative real-time PCR
SNPs: Single nucleotide polymorphisms
MDA: Malondialdehyde
SOD: Superoxide dismutase
GSH: Glutataione
POD: Peroxidase
