# Supplementary figures and images for: Asymmetric expression of homoeologous genes contributes to dietary adaption of an allodiploid hybrid fish derived from Megalobrama amblycephala (♀) × Culter alburnus (♂)
Source: BMC Genomics. 2021 May 19;22:362. doi: 10.1186/s12864-021-07639-6 (PMC8132401; doi:10.1186/s12864-021-07639-6)

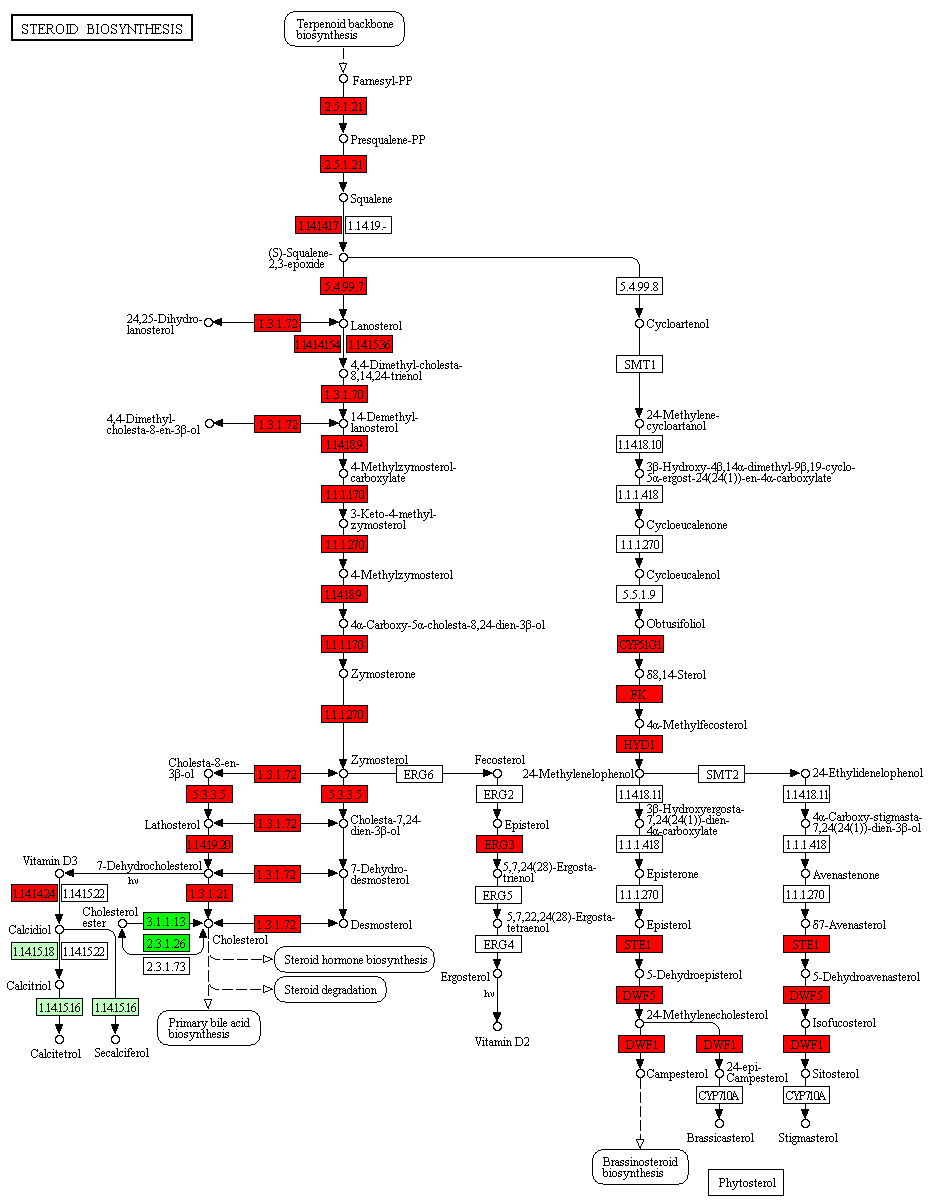

Supplement: Supplementary file 6 — Additional file 6: Figure S1. Steroid biosynthesis pathway and the differentilly expressed homoeologous genes in the herbivorous diet group compared with carnivorous diet group. Genes in red boxes were upregulated, and those in green boxes were downregulated. [file 12864_2021_7639_MOESM6_ESM.tif]

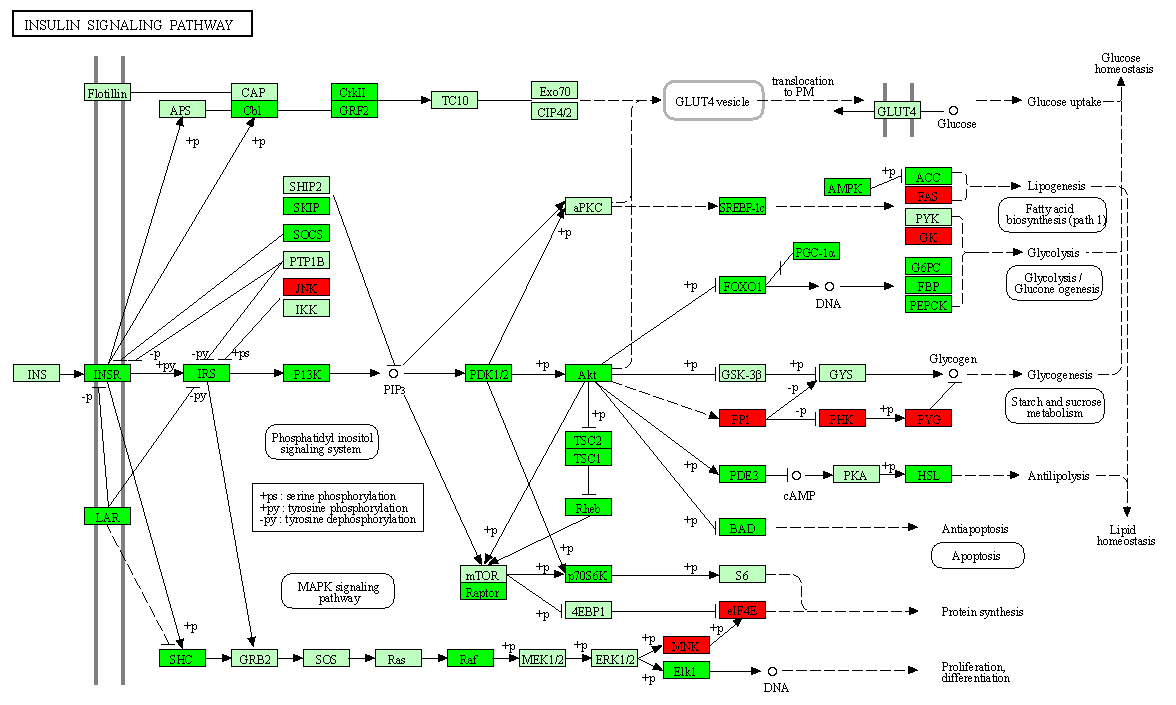

Supplement: Supplementary file 7 — Additional file 7: Figure S2. Insulin signaling pathway and the differentilly expressed homoeologous genes in the herbivorous diet group compared with carnivorous diet group. Genes in red boxes were upregulated, and those in green boxes were downregulated. [file 12864_2021_7639_MOESM7_ESM.tif]
