# Supplementary material for: Asymmetric expression of homoeologous genes contributes to dietary adaption of an allodiploid hybrid fish derived from Megalobrama amblycephala (♀) × Culter alburnus (♂)
Source: BMC Genomics. 2021 May 19;22:362. doi: 10.1186/s12864-021-07639-6 (PMC8132401; doi:10.1186/s12864-021-07639-6)
Supplement: Supplementary file 8 — Additional file 8. Supplement tables (Table S1, Table S2 and Table S3). [file 12864_2021_7639_MOESM8_ESM.docx]

Table S1 Special expressed genes in herbivorous compared with carnivorous diet fish

| Liver tissue | | | Intestine tissue | | |
| --- | --- | --- | --- | --- | --- |
| Gene symbol | Log2(fold change) | KO ID | Gene symbol | Log2(fold change) | KO ID |
| *sqlea* | 9.59 | K00511 | *cyp7a1* | 8.30 | K00489 |
| *mcm6* | 5.86 | K02542 | *sqlea* | 6.75 | K00511 |
| *ntd5* | 5.76 | K17305 | *col12a1* | 5.64 | K08132 |
| *a2m* | 5.47 | K03910 | *rpe65b* | 5.39 | K20991 |
| *epoa* | 5.19 | K05437 | *clca5.2* | 4.98 | K05030 |
| *hells* | 5.17 | K19001 | *gamma 3* | 4.95 | K06247 |
| *mcm3* | 5.16 | K02541 | *rbm4.1* | 4.76 | K13187 |
| *dtx1* | 4.98 | K06058 | *itga11a* | 4.75 | K06587 |
| *fd16g01* | 4.74 | K16769 | *guca1b* | 4.69 | K08328 |
| *cdc20* | 4.71 | K03363 | *vcanb* | 4.40 | K06794 |
| *lhx9* | 4.68 | K09373 | *kcnj15* | 4.36 | K05008 |
| *chaf1a* | 4.63 | K10750 |  |  |  |
| *dnajc6* | 4.58 | K09526 |  |  |  |
| *hpgd* | 4.41 | K00069 |  |  |  |
| *kcnh5a* | 4.36 | K04908 |  |  |  |
| *uhrf1* | 4.32 | K10638 |  |  |  |
| *fkrp* | 4.07 | K19873 |  |  |  |

Table S2 Summary of the differentially expressed allelics in intestine and liver tissues

|  | Higher expression gene NO. | | | Lower expression gene NO. | | | Total |
| --- | --- | --- | --- | --- | --- | --- | --- |
| Gene original | TC | BSB | New gene | TC | BSB | New gene |  |
| H-I_Vs_C-I | 1180 | 471 | 795 | 1464 | 459 | 463 | 4832 |
| C-L_vs_H-L | 762 | 180 | 302 | 717 | 247 | 250 | 2538 |

Table S3 Primers of the twelve differeninally expressed allelics for qPCR

| Genes | Forward primer | Reverse primer | PCR length |
| --- | --- | --- | --- |
| *hmgcra* | ACAAGCCTAACCCTGTCACG | GGCATGTCTGGCTCAATCCT | 178 |
| *gck* | CTGCTGAAAACACGTGGAGC | GACGGCAAGATTCCCAGTGA | 113 |
| *ebp* | TGAACCACAATGTACCGCGA | ATCCTCTTCTGCAGCCGTTC | 187 |
| *idi1* | CACCTGTAAGGATGCCGGAG | GTCGTCGATCAGGATGCACA | 89 |
| *cyp51* | GCGCTCATGTTCAACAGCAA | TGTTCAGTCCGGTCTTCAGC | 151 |
| *mvd* | GCCGGGCCTAATGCTGTTAT | TGTATGCCCTTTGGAGTGG | 187 |
| *mat1a* | CGCTGGGTGGCTAAATCTCT | GATTGAAAGAGGGTGGGCGA | 96 |
| *lipg* | CAGAGACGAGAATCCGAGGC | GGTTGACGGCATCAGGGTAA | 82 |
| *pck1* | ATCCCAACTCTCGCTTCTGC | TTCAGATCTCATCGCGGCTC | 188 |
| *ulk* | GTTCTGGAGTCAGTGGACCG | ATAAGGCGTGTGGCTGTCTC | 119 |
| *doi3* | CTTACCGCGATTTCTCGTCG | CACAGCGGAGGGTCTTCATC | 120 |
| *bco1* | AACAGCCCTTGAAGCTGGAT | GCCATCGCACCAGTGTAGAA | 166 |
